# The Association Between Emotional Expressions and Empathic Accuracy

**DOI:** 10.21203/rs.3.rs-3858053/v1

**Published:** 2024-01-16

**Authors:** Tong Lin, Jeremy C. Simon, Jennifer N. Gutsell

**Affiliations:** Brandeis University; Museum of Science; Brandeis University

**Keywords:** Empathy, empathic accuracy, dyadic interaction, facial expression, emotional experiences

## Abstract

Empathic accuracy, the ability to accurately represent and understand another’s emotional state, is integral to socio-emotional functioning. It is also inherently an interpersonal process that requires active engagement of the emotional systems of both interaction partners. The emotional expressivity of the partner sharing their emotions restricts empathic accuracy and the perceiver’s emotional expressivity might also affect empathic accuracy as they behaviorally simulate and thus share the emotions they see in the other’s face. We explored a potential role of emotional expressivity in people’s ability to understand another’s emotions in a face-to-face dyadic interaction. Participants took turns sharing emotional experiences while their facial expressions were recorded. They then watched the recordings while continuously rating their own and their partner’s affect at any given point during the recording. Empathic accuracy was indexed as the epoch by-epoch emotion change detection. We found that emotional expressivity of the listener, but not of the partner, was associated with increased empathic accuracy, even when controlling for partner’s expressivity. Our findings highlight the active role the person empathizing takes in face-to-face emotional sharing.

## Introduction

Empathic accuracy, the ability to accurately represent and understand another’s emotional state (Hess & Blairy, 2001) plays a crucial role in navigating the social world: deficits in empathic accuracy can impair social functioning, as it is associated with lower relationship satisfaction (Sened et al., 2017), worse conflict resolution (Bates & Samp, 2011), less responsive behavior in dyadic interaction (Winczewski et al., 2016), and worse therapy outcomes (Atzil-Slonim et al., 2019).

Empathic accuracy in real life includes at least two interaction partners, as empathy is an inherently social phenomenon (Ickes et al., 1990). It is, thus, crucial to study empathy in a dyadic setting that allows enactment of real-life social exchange and that can capture the interpersonal nature of empathy (Mackes et al., 2018). Recent work has started to investigate empathy in face-to-face dyadic interactions (e.g. Brown et al., 2020; Simon & Gutsell, 2021; Thompson et al., 2022). The current study aims to contribute to this growing body of dyadic empathic accuracy research by looking at the role of emotion expressions.

In face-to-face empathy, emotional expressions of both interaction partners might contribute to empathic accuracy. Communicators vary in regards to how much of their emotional experience they express outwardly (Burgin et al., 2012; Gross & John, 1997) and they also might modulate their expression for communicative purposes (Fridlund, 2014; Kappas & DescÔteaux, 2003). At the same time, perceivers do not merely recognize emotions from nonverbal displays. They also respond to them with spontaneous expressions of their own emotional state, mimicry and communicative expressions that range from tenderness to antagonism and avoidance (Keltner et al., 2019). There is initial evidence that the emotional expressivity of both interaction partners is important for empathic sharing, as higher trait empathy is only associated with better empathic accuracy when the person sharing their emotions is generally emotionally expressive (Zaki et al., 2008). Moreover, the lack of emotional expressions of an interaction partner results in an elevated stress response in the person sharing (Kouzakova et al., 2010). Additionally, people with higher levels of emotional expressivity derive greater pleasure from social interactions (Burgin et al., 2012) and lower levels of emotional expressivity have been linked to social anhedonia (Leung et al., 2010) depression (Sloan et al., 2001), schizophrenia (Earnst & Kring, 1999), and other psychological impairments (Watson et al., 1984). Specifically, the more expressive people report they are, the more likely they are to report that they experience these emotions more strongly (Kring et al., 1994). Interaction partners’ facial expressions may also be a result of mimicry – the mirroring of facial expressions, which is an important aspect of empathic responding (Lamm et al., 2008; Singer & Lamm, 2009; Walter, 2012) according to a meta-analysis (Holland et al., 2021).

In sum, emotional expressivity of both interaction partners might affect empathy and empathic accuracy. However, how emotional expressivity of two interaction partners individually and jointly might relate to empathic accuracy is unknown. Hence the current study investigates the link between facial expressions of both interaction partners and empathic accuracy in a realistic interaction setting.

Characteristics of empathy can be divided into three categories: (1) experience sharing: vicariously sharing targets’ internal states, (2) mentalizing: understanding the targets’ mental states, and (3) prosocial concern: expressing motivation to improve targets’ experiences (Zaki & Ochsner, 2012). According to the perception-action model (PAM) of empathy, at least the experience sharing aspect of empathy results from the automatically activated mental representations of another’s motor behavior in an observer’s mind when they pay attention to the other’s emotional state (Preston, 2007). Hence perceiving and generating actions share some mental representations (Preston, 2007), thus closely linking emotional expressions in response to others’ perceived emotional state with empathy.

Emotional expressivity of the person who is sharing an emotional experience might have an impact on empathic accuracy as previous studies suggest that individuals tend to empathize with targets who are more emotionally expressive (Lee et al., 2011; Zaki et al., 2009). However, these studies examined emotional expressivity as a trait instead of measuring the actual emotional expressivity the sharer displayed during the experiments. Using a behavior-based method to measure emotional expressivity is important because trait expressivity from self-report assessments might differ from moment-to-moment state expressivity in different situational context. In the current study, we examined how sharers’ emotional expressivity influences perceivers’ empathic accuracy during *in vivo* interaction.

The emotional expressivity of the listener might also have an impact on empathic accuracy through mimicry, which refers to spontaneous facial muscular activity that a person displays when they are presented with another individual’s facial expressions (Hess et al., 1999). Mimicry potentially allows us to share other individuals’ emotions because subjective emotional experience is affected by the feedback from facial mimicry (Hatfield et al., 1993). When observing emotional expressions in others, people unconsciously mimic their expressions (Dimberg et al., 2000). Interestingly, interfering with mimicry also interferes with the ability to recognize emotional expressions at least for highly empathic individuals (Jospe et al., 2018). As these previous studies suggest, people’s sensitivity in reacting with facial reactions to facial expressions is related to empathic accuracy (Dimberg et al., 2011) and mimicry can indicate experience sharing in the empathic process (Hess et al., 2017). A recent meta-analysis revealed that mimicry is associated with trait empathy (Holland et al., 2021), but the relationship between mirroring of facial expressions and empathic accuracy is unclear, as some work shows a positive association (Achaibou et al., 2008) while other work shows no effect (Holland et al., 2021).

Emotional expressivity apart from mimicry may also enhance the emotional experience by influencing emotion co-regulation and communication quality. According to the social baseline theory, social connections are fundamental to emotional regulation (Beckes & Coan, 2011a). People engage with others’ emotions by responding and modulating their emotional response. Co-regulation can occur through nonverbal cues including facial expressions (Beckes & Coan, 2011b). When people suppress their facial expressions, they engage in a negative form of emotion regulation that involves hiding or concealing one’s emotions (Butler et al., 2003) and such expressive suppression increased stress in both themselves and their partners, and also disrupted communication (Butler et al., 2003).

Affiliative emotional expressions as well can foster feelings of closeness and affiliation, which can contribute to empathic accuracy. Facial expressions can convey complicated social information. For example, the expression of pride may communicate an individual’s social status and group acceptance (Tracy & Robins, 2007). When individuals express their emotions, it can elicit emotional responses in the receivers and hence improve communication quality. For example, individuals who received a genuine smile from a stranger experience more pleasure and empathy than those who received a noun-genuine smile (Surakka & Hietanen, 1998).

## The current study

The goal of the current study was to investigate how embodied cognition and the motivation to affiliate might influence people’s ability to understand others’ emotions through facial expressions. Specifically, the current study examined the influence of facial expressions on empathic accuracy in a face-to-face experience sharing task. Experience sharing is usually examined through self-report (Ali et al., 2009; Atkins et al., 2016; Clark et al., 2008) and empathy targets are usually depicted as images or videos rather than actual interaction partners, which lack in realism. Therefore, we aimed to examine emotional sharing in a naturalistic setting. We predicted that having more emotion expressions would be associated with increased empathic accuracy. To investigate the potential contribution of emotional expressivity of both empathizer and empathy target in a face-to-face interaction, participants engaged in face-to-face experience sharing while also being video recorded. The resulting videos provided the basis for behavioral coding of emotional expressivity of both the sharer and the listener. We preregistered (https://archive.org/details/osf-registrations-vsybe-v1) our hypotheses and the main analyses and mentioned deviations from the preregistration in the results section. Study materials and data can be found on the Open Science Framework (OSF; https://osf.io/v25u8/? view_only=c03337a6dde04ce894bca9c7fc955747)

## Methods

### Participants

We recruited 76 participants in total. Participants were rejected due to malfunctioning equipment (N = 4), missing data including missing their partner’s data (N = 18). Due to the dyadic design, if one participant was missing one data point, the whole dyad was removed from the analysis because both interacting partners’ data were needed for each of their analysis. The final sample consisted of 54 participants (Mean age = 37.2 years (SD = 17.8) ranging from 18 to 70 years old; 27 females, 25 males, and 2 identified as “other”; 9 African Americans, 44 European Americans, and 1 identified as “others”). Participants provided informed consent prior to participation and the study was approved by our Institutional Review Board. Please note that the current study is part of a larger study that also looked at EEG indicators of experience sharing, which is covered under the same pre-registration1. Based on power simulations (with 1000 replications, ICC = 0.1) using SIMR package in R, expected to need 45 dyads to achieve 80% power to detect a medium effect (r = .4). Our final sample of 27 dyads provides an 71% actual power to detect an effect size of 0.018 for listeners’ facial expressions based on 1000 simulations (α = .05).

### Procedures

**Figure 1:**
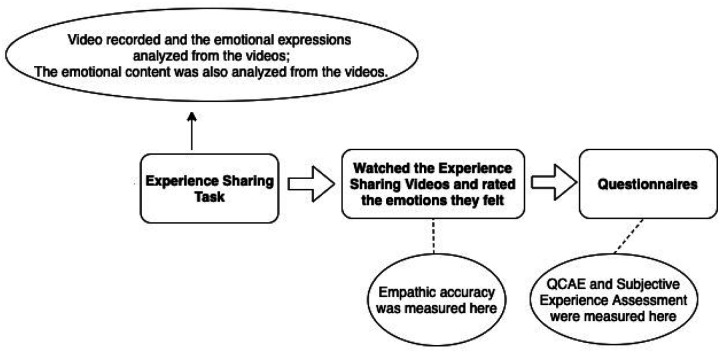
Procedures of the Experiment

Participants were run in pairs in a single experimental session that lasted about three hours (please see Fig. 1 for a diagram of the procedure). All instruments and measures referred to here are described in detail in the next section. After arriving in the laboratory separately, participants were placed in separate rooms, were fitted with EEG caps for electrophysiological recordings, and completed a series of baseline measures unrelated to the current study. Following they were introduced to each other and went through an ice breaker exercise where they took turns responding to 5 small talk prompts in order to get familiar with each other. They then completed a couple of tasks together including an experience sharing task that served as the basis for our empathic accuracy and emotional expressivity measures. While the dyads were talking and listening, both partner’s facial expressions were videotaped. Finally, participants were separated again to complete empathic accuracy ratings and a series of self-report measures about the interaction experience. Only the tasks relevant to this current study will be described in detail here.^2^

## Materials & Measures

### Experience Sharing Task

Participants were told that they would be sharing two experiences with their partner: one positive experience and one negative experience. Specifically, they were told to “take a moment to think of a most positive/negative personal event that [they] would like to share” and “try to choose an event which [they] can speak about for a full 2 minutes, but no longer”. Before experience sharing, participants were asked to recount a neutral event (how they got to the laboratory that day) to familiarize themselves with the paradigm. Listeners were instructed not to talk during the experience sharing but were allowed to react non-verbally such as smiling in order to make the interaction feel more natural. Experimenters separately video recorded both the sharer and the observer during this experience sharing task.

### Empathic Accuracy Task (adapted from Zaki et al., 2008)

Participants watched their partner and their own videos after the experience sharing task. While watching the video, they rated the emotions of their partner and themselves on a rating bar of a 9-point Likert scale from “very negative” to “very positive” through arrow keys: clicking the left arrow would mean feeling more negative emotions and clicking the right arrow would indicate more positive emotion.

We followed how previous studies calculated empathic accuracy: we operationalized empathic accuracy by quantifying participants’ agreement on how emotional content of the video changes (Genzer et al., 2022) For each one-second interval, we classified participants’ ratings into one of three categories: either an increase in affect, a decrease, or maintained from the previous epoch. Specifically, we de ned deltaPart(t) as a scale-invariant derivative:

deltaPart(t) = {“increase”, if part(t) - part(t-1) > 0 {“maintain”, if part(t) - part(t-1) = = 0 {“decrease”, if part(t) - part(t-1) < 0 We then defined a corresponding variable, deltaTarg(t), using the target’s self-reported ratings (Genzer et al., 2022). We then operationalized change detection such that a “successful” change detection occurs if participants’ rated change matches the target’s change at that second. If they did not match, it was labeled a “failed” change detection (Genzer et al., 2022).

changeDetection(t) = {1, if deltaPart(t) = = deltaTarg(t) { 0, otherwise

Therefore, change detection was a binary variable for each one-second interval that reflects whether the participant successfully detected any change in the target’s affect (Genzer et al., 2022). Last, we averaged across the entire period of each shared experience, so the final score ranged from 0 to 1, with 1 reflecting the greatest empathic accuracy. Then we created averages of the positive and the negative experience for each participant, so the final score for empathic accuracy was a continuous variable ranging from 0 to 1.^3^

## Expressivity of the Emotional Sharing

For each video of experience sharing, the sharer’s facial expressions were coded as the sharer’s expressivity and the listener’s facial expressions were coded as the listener’s expressivity. Both sharers and listeners in both positive sharing condition and negative sharing condition were coded by two research assistants using *The Facial Expression Coding System (FACES)* (Kring & Sloan, 2007). The subcomponents of facial expression coding included frequencies, duration, intensities, and valence of the expressions. Raters were trained to use the FACES.

When raters detected a change in the participant’s facial expression on the video, they wrote down when this change occurred and ended, as well as its intensity and valence (either positive or negative). The minimum rating for frequency, duration and intensity would be zero and there would be no upper limit. Following previous studies that used this scale (Kring & Gordon, 1998; Kring & Sloan, 2007), we only used frequency, how often an emotional expression occurs, to reduce the number of dependent variables in the analysis. The average for frequency was 2.85 (SD = 2.43). The other two components were strongly correlated with frequency when we tested the subscales (all rs > 0.8, ps < .001, n = 66). We used the total frequency of positive expressions during the positive condition as an index of each participant’s positive emotional expressions, and the total frequency of negative expressions during the negative condition as an index of each participant’s negative emotional expressions. We then averaged the frequencies of both positive and negative facial expressions in both positive and negative conditions to calculate the average emotional expressivity of each participant. The Intraclass Correlation Coefficient (ICC) between raters was higher than 0.75 to ensure adequate inter-rater reliability as shown in Table 1.

The *QCAE (Questionnaire of Cognitive and Affective Empathy)* (Reniers et al., 2011): Participants filled out an QCAE that measured their trait empathy on a 4-point likert scale (“strongly agree”, “slightly agree”, “slightly disagree”, “strongly disagree”) based on 5 subscales which are (1) Perspective taking, (2) Online simulation, (3) Emotion contagion, (4) Proximal responsivity and (5) Peripheral responsivity subscale. Cognitive empathy was calculated based on the average of items in “Perspective taking” and “Online simulation” subscales. Affective empathy was the average of items in Emotion contagion.

### Subjective Experience Assessment

After completion of the empathic accuracy measure, participants completed a series of self-report measures of subjective experience of the interaction: Their desire to affiliate and their positive evaluations (Holoien et al., 2015). Specifically, participants answered ten items taken from Holoien, Bergsieker, Shelton, and Alegre (2015) indexing their desire to affiliate with their partners (“How much did you want to get along with your partner?”; “How much did you want to have a smooth interaction with your partner?”; “How much did you like your partner?”) and how positive their impressions were of their partners (“Based on your interaction with your partner, how pleasant/warm/likable/natural/confident/attractive/intelligent did he or she seem?”).

### Emotional Content of the Conversation

To assess affective content of the shared emotional experiences, we utilized Sentiment Analysis using Stanford CoreNLP version 3.8.0 (Manning et al., 2014) and Sonix (*Automatically Convert Audio and Video to Text*, n.d.), an online automated transcription software to convert audio files (.MP4) of participants telling positive and negative experiences to text files, with an average accuracy above 85%. Specifically, Sonix transcribed all videos’ audio into texts and they were all visually inspected by research assistants for mistakes. Then the Stanford CoreNLP’s sentiment analysis employed a sophisticated approach that began with tokenizing the text and analyzing its grammatical structure (Manning et al., 2014). A grammatical tree structure was then constructed to represent the relationships between words, and sentiment scores were assigned to various parts of the sentence (Manning et al., 2014). These scores were then aggregated to yield an overall sentiment score for the passage, which was categorized into sentiment classes “very negative”, “negative”, “neutral”, “positive” and “very positive”. For the current study, the combined percentages from the “very negative” and “negative” categories indicate the negative content, and combined percentages from the “very positive” and “positive” categories indicate the positive content. The overall emotional content of each video was calculated using the average of the positive content and the negative content.

## Results

We tested simple correlations for all covariates in the multiple regression model, which can be found in the correlation table (Table 2).

### Hypothesis Testing

We used multi-level modeling for all our hypotheses to account for the dyadic nature of our data. To test the hypothesis that more emotional expressivity was related to better empathic accuracy we conducted a 2-level multilevel model where the listener expressivity scores were nested within dyads

*Model1* Level1: Estimated Empathic Accuracy=β0_i_ + β1_i_ Expressivity+ ϵi

Level2: β0_i_ = γ_00_ + U_0i_

β1_i_ = γ_10_ + U_1i_

**Figure 2:**
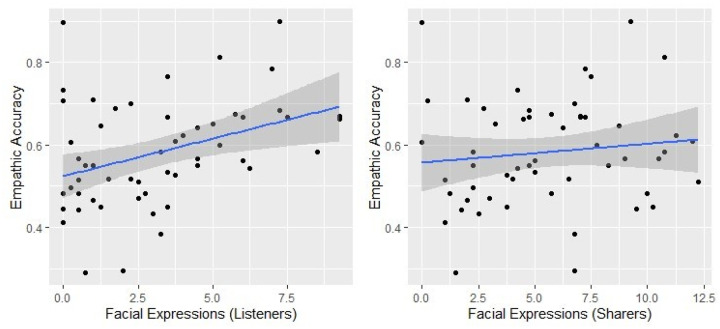
The Scatterplot of emotional expressivity and emotional accuracy with a regression line

We hypothesized that participants would empathize with their partners more effectively when listeners displayed more facial expressions, so we expected β1 to be significant. We found a significant relationship between listeners’ expressivity and empathic accuracy supporting our first hypothesis, β1_i_ (listener expressivity) = 0.37 *p* = .006. We also hypothesized that participants would empathize with their partners more effectively when their partners display more emotional facial expressions. The results did not show a statistically significant relationship between the sharer’s expressivity and empathic accuracy, β1_i_ (sharer expressivity) = 0.1, *p* = 0.489. Controlling for the sharer’s expressivity, listener’s expressivity still was positively related to empathic accuracy, β1i (listener expressivity) = 0.38, *p* = 0.009, suggesting that listener expressivity uniquely contributes to empathic accuracy. This pattern can also be seen on the scatterplot in Figure 2.

We also explored several possible additional confounds that might influence empathic accuracy. We tested for potential effects of the desire to affiliate with the partner, the personal evaluation for the partner, trait empathy including both affective and cognitive empathy, and the emotional content of the conversation in a multiple linear regression:

#### Full Model

Level1: Estimated Empathic Accuracy=β0_i_ + β1_i_ Expressivity(Listeners)+ β2_i_ Expressivity(Speakers)+ β3_i_ Desire_+_ β4_i_ Evaluation+β5_i_ Cognitive Empathy+ β6_i_ Affective Empathy + β7_i_ Content+ ϵi

Level2: β0_i_ = γ_00_ + U_0i_

β1_i_ = γ_10_ + U_1_

Expressivity predicts empathic accuracy, even when controlling for these other variables, (β1_i_ = 0.4 *p*< .01; β2_i_ = 0.04, *p*= 0.81; β3_i_ = −0.02, *p*= 0.91; β4_i_ = −0.14, *p*= 0.36; β5_i_ < .01, *p*= 0.97; β6_i_ = −0.04, *p*= 0.80; β7_i_ = 0.09 p= 0.52).

## Discussion

Empathic accuracy is fundamental to effective navigation of the social world. Because empathy is an inherently interpersonal process, both partners are likely to contribute to successful empathy. Here we investigated how emotional expressivity of two interaction partners individually and jointly contribute to empathic accuracy in a naturalistic face-to-face interaction.

Our results support the hypothesis that displaying more emotional expressions when listening to another’s emotional experiences is related to increased empathic accuracy. This result indicates that actively responding to another induvial with facial expressions during a social interaction might influence one’s own ability of empathizing with the other – an idea supported by the embodied cognition theory that the mind is not just a product of the brain’s neural activity but also shaped by motor actions and the body’s interactions with the world (Foglia & Wilson, 2013; Fuchs, 2009).

## The role of the listener’s emotional expressivity

The exact mechanism underlying how Listener’s expressivity is associated with empathic accuracy is still unclear as emotional expression serve multiple purposes. Observers’ mimicry could induce a feedback process, leading the observer to experience the emotion they mimicked (Helt et al., 2010; Prochazkova & Kret, 2017; Tia et al., 2011)). Our measure of emotional expressivity was coded for each individual participant and not the dyad, so whether the expressed emotions of the listener matched those of the sharer is unknown. Given previous findings linking mimicry to empathic accuracy (Hess & Blairy, 2001), facial feedback resulting from facial mimicry is a likely contributor to our findings.

Another possible explanation is consistent with the communicative role of emotional expressions. During an interaction, partners might express affiliative emotions or mimic each other’s emotion expression in order to promote mutual liking (Kulesza et al., 2015; Salazar Kämpf et al., 2018). Affiliation along with empathy help individuals to maintain social bonds (Seyfarth & Cheney, 2013) and empathic accuracy has also been suggested to carry advantages for maintaining social bonds (Soto & Levenson, 2009). In our study, the association between listener expressivity and empathic accuracy, held even when controlling for the listeners’ desire to affiliate with their interaction partner and the extent to how much they liked their partner. Thus, affiliative emotion expressions might contribute to empathic accuracy but is unlikely to be the main explanation for our findings.

The listener could also communicate specific emotions in order to influence the sharer’s emotions (Bavelas et al., 1986). Such emotional co-regulation could theoretically lead to increased empathic accuracy since the listener contributed to the target’s emotional state. However, such a connection between emotion co-regulation and empathic accuracy has yet to be established and our data is insufficient to test this hypothesis. Finally, the listener’s emotional expressions could be reflections of their own emotional state which might match (e.g. responding with sadness to the sharer’s sadness, be complementary, or responding with warm feelings of tenderhearted sympathy to the sharer’s sadness). Hence, listener emotional expressivity might be indicative of empathy or sympathy (Hess & Fischer, 2013). Unfortunately, we did not include a state empathy measure or a measure of the listeners own emotional state in our study, but such measures would be important to include in future work.

## The role of the sharer’s emotional expressivity

Contrary to our predictions, the sharers’ expressivity did not have a statistically significant impact on empathic accuracy. This is contrary to previous findings showing that target trait level emotional expressivity limits the influence of listener’s trait empathy on listeners’ empathic accuracy (Zaki et al., 2008). A potential reason for this discrepancy in findings could be that the previous work assessed self-reported trait emotional expressivity while the current study uses coded facial expressions. It could also be that sharers’ expressivity plays less of a role in face-to-face interactions as opposed to when listeners simply watch videos of sharers sharing their experiences. During interactions, the shared story has context and so emotions can be inferred. The listener might infer the sharer’s emotional state from a complex set of information using cognitive empathy, not just reading their partners’ emotional expressions. Indeed, empathic accuracy has been shown to be related to cognitive empathy but unrelated to affective empathy (Mackes, Golm, O’Daly, et al., 2018) or sometimes even negatively correlated with experience sharing (Kral et al., 2017).

Another potential explanation for finding effects for listeners but not for sharers could be attributed to the unfamiliarity between the interacting individuals. People might be more guarded in their expressions when sharing an emotional story with a stranger than they would be with someone they know as previous literature shows that the degree to which emotions are expressed depends on the familiarity of an accompanying person (Wagner & Smith, 1991; Zhang & Parmley, 2011). Whereas, listeners might instead feel they should be expressive to make the stranger feel validated. Supporting this notion, prior research has demonstrated that close friends exhibit greater accuracy in interpreting subtle expressions of emotions like sadness and happiness, compared to mere acquaintances (Zhang & Parmley, 2011).

## Methodological Considerations

### Limitations and Future Directions

The present findings suggested that there is a relationship between facial expressions and understanding others’ emotions in face-to-face interactions. They are in line with the facial feedback hypothesis and also contribute to our understanding of facial mimicry (Dimberg et al., 2000; Gump & Kulik, 1997; Lundqvist, 1995) that also has other affiliative functions (Fridlund, 1994; Hess et al., 1995), which are not applicable in non-dyadic experimental setups. However, it should also be noted that our study was not designed to differentiate whether the observed effects were due to listener expressivity or listener mimicry. Different expressions might signal different intentions (e.g., sadness vs. happiness) and different social functions of mimicry are suggested depending on the expression that is mimicked (Fischer & Hess, 2017; Prochazkova & Kret, 2017). Although it could be theorized that the observed association between listener expressivity and empathic accuracy could be due to an underlying process of emotional mimicry, further research is needed to disentangle mimicry from expressivity in order to support or refute this theory.

The behavioral coding in our study might miss nuances. This is because we found relatively low numbers of emotion expressions and large variability between individuals. It might be useful to employ facial electromyography (EMG) to capture subtle mimicry that has been found to be related to the perception of others’ emotions and other aspects of social perception (e.g., Sato et al., 2013). It might also be useful to increase sample sizes in future studies to be able to differentiate between different emotions and to directly measure facial muscle movements instead of coding expressions. Moreover, this might increase the likelihood to find additional effects or interaction effects between expressivity and other factors like the valence of the emotional account of the emotional experiences, listener empathy, or listener’s desire to affiliate with the sharer (e.g., Neumann et al., 2015; Oberman et al., 2007).

Another limitation of this study is the potential impact of the EEG equipment on participants’ mobility, which may have constrained their ability to express facial emotions effectively. This raises the possibility that, in the absence of such restrictions, the expressivity of the sharers might have been more pronounced. It is noteworthy, however, that despite these limitations in movement, significant effects on the expressivity of listeners were still observed. Additionally, the prohibition of verbal responses from listeners might be a methodological constraint. It would be beneficial for future research to replicate these findings in settings that allow for even more natural interactions such as utilizing less cumbersome EEG equipment and enabling listeners to engage verbally.

Our study had the advantage to have realistic interactions instead of stripped-down stimuli like images or short videos. For instance, in real life, empathic accuracy usually entails a mixture of visual and verbal information as well as context information. Verbal information has been found to contribute more to empathic accuracy than visual information, but a combination of both seem to produce the highest empathic accuracy (Zaki et al., 2009; Jospe et al., 2020). We used sentiment analysis to include verbal information in the current study (Korhonen et al., 2002; Mohammad, 2012; Skerry & Saxe, 2015) and correlated this with empathic accuracy, but we only assessed words that were spoken. Furthermore, our research approach captured the natural flow of communication between individuals, not just the expressions and actions of isolated individuals. Thus, the approach was closer to real life and our approach might help to identify dyadic effects that cannot be found in non-dyadic experiments. Our findings that empathic accuracy is associated with listener expressivity might be just such a finding.

### Conclusions

In conclusion, we find that one’s emotional expressivity, specifically the act of displaying more emotional expressions when listening to another’s emotional experiences, is related to empathic accuracy. Further research is needed to fully understand how emotional expressivity may affect empathy in real-world scenarios, to investigate whether there is a causal connection and to determine if this relationship is mediated by other factors like affiliative motivations, mimicry, or emotion co-regulation. It will be important to refine the methodology in future research to investigate the impact of subtle emotion expressions on empathic accuracy in actual interactions and to extend research to the role of body language in empathic accuracy (e.g. Moore et al., 2012).

### Declarations

All authors certify that they have no affiliations with or involvement in any organization or entity with any financial interest or non-financial interest in the subject matter or materials discussed in this manuscript.

## Figures and Tables

**Table 1:** Inter-rater Reliability Table

	Listeners	Speakers
Positive	0.906	0.871
Negative	0.766	0.841

**Table 2:** Correlation Table for all Covariates

	Desire To Affiliate	Evaluation	Cognitive Empathy	Affective Empathy	Listener Expression	Sharer Expression	Empathic Accuracy	Emotional Content
Desire To Affiliate	1.000							
Evaluation	0.583***	1.000						
Cognitive Empathy	0.183	0.121	1.000					
Affective Empathy	0.381**	0.083	0.225	1.000				
Listener Expression	0.132	0.219	0.105	0.148	1.000			
Sharer Expression	0.208	0.285*	0299*	0.177	0.375**	1.000		
Empathic Accuracy	−0.064	−0.063	0.030	−0.014	0.382**	0.118	1.000	
Emotional Content	−0.157	−0.157	−0.044	−0.152	−0.159	−0.219	0.041	1.000

Note:

* p<0.05,

** p< 0.01,

*** p<0.0001

The numbers are correlation coefficients

## Footnotes

2. In addition to the tasks described here, the participants completed a ball squeezing task, during which they took turns repeatedly squeezing and observing their partner squeezing a stress ball. In 50% of the cases they also completed an action coordination task before experience sharing. For the other 50% they did the action coordination after experience sharing but before the measurement of empathic accuracy. All dyads also did another round of ball squeezing before they were separated into individual testing rooms. A subset of these additional measures have been published in Simon & Gutsell (2021).

3. Since the more commonly applied measure of empathic accuracy for this task is to calculate the time-course correlation between target and empathizer’s responses, we checked whether results would differ using this method. The two empathic accuracy scores were correlated r = 0.28, p = 0.04 and the hypothesis testing results do not differ using the time-course correlation measure of empathic accuracy: Estimated Empathic Accuracy = β0_i_ + β1_i_ Expressivity+ ϵi β1_i_ (listener expressivity) = 0.37, p = .006; β1_i_ (sharer expressivity) = 0.2, *p* = 0.16; Controlling for the sharer’s expressivity, listener’s expressivity still was positively related to empathic accuracy, β1i (listener expressivity) = 0.35, *p* = 0.017
